# Effects of Solidification Cooling Rate on the Microstructure and Mechanical Properties of a Cast Al-Si-Cu-Mg-Ni Piston Alloy

**DOI:** 10.3390/ma11071230

**Published:** 2018-07-18

**Authors:** Lusha Tian, Yongchun Guo, Jianping Li, Feng Xia, Minxian Liang, Yaping Bai

**Affiliations:** Materials and Chemical Engineering School, Xi’an Technological University, Xi’an 710021, China; tianlusha51@163.com (L.T.); yc-guo@163.com (Y.G.); xiafeng0811@163.com (F.X.); dlmxian@163.com (M.L.); jingpingxue2004@163.com (Y.B.)

**Keywords:** Al-13Si-4Cu-1Mg-2Ni alloy, cooling rate effect, solidification microstructure, second phase, mechanical property

## Abstract

The effects of cooling rate 0.15, 1.5, 15, 150, and 1.5 × 105 °C/s on the microstructures and mechanical properties of Al-13Si-4Cu-1Mg-2Ni cast piston alloy were investigated. The results show that with an increase of solidification cooling rate, the secondary dendrite arm spacing (SDAS) of this model alloy can be calculated using the formula D = 47.126v − 1/3. The phases formed during the solidification with lower cooling rates primarily consist of eutectic silicon, M-Mg_2_Si phase, γ-Al_7_Cu_4_Ni phase, δ-Al_3_CuNi phase, ε-Al_3_Ni phase, and Q-Al_5_Cu_2_Mg_8_Si_6_ phase. With the increase in the solidification cooling rate from 0.15 to 15 °C/s, the hardness increased from 80.9 to 125.7 HB, the room temperature tensile strength enhanced from 189.3 to 282.5 MPa, and the elongation at break increased from 1.6% to 2.8%. The ε -Al_3_Ni phase disappears in the alloy and the Q phase emerges. The δ phase and the γ phase change from large-sized meshes and clusters to smaller meshes and Chinese script patterns. Further increase in the cooling rate leads to the micro hardness increasing gradually from 131.2 to 195.6 HV and the alloy solidifying into a uniform structure and forming nanocrystals.

## 1. Introduction

Piston, known as the “heart” of the engine, is a key component of internal combustion engine. Operating in a harsh environment, piston endure high temperature, high pressure, complex friction, and coupled thermo-mechanical load. With the improvement of engine power density (kW/L), demanding requirements imposed on the piston material increased greatly [1,2,3,4].

Among various piston alloys, near-eutectic Al-Si-Cu-Mg-Ni alloys, involving Al-13Si-4Cu-1Mg-2Ni alloy, were widely used in high performance diesel engine piston since the near-eutectic alloys have a better combination of light weight, high mechanical properties and a better castability combination than other alloys. Up to date, intensive studies have been made on the heat treatment process and the microscopic thermal characteristics of the Al-13Si-4Cu-1Mg-2Ni alloy [5]. Yongchun Guo, Tao Xu et al. studied the precipitation phase in the Al-Si-Cu-Mg-Ni piston alloy and analyzes the five kinds of Al-13Si-1Mg-2Ni-xCu (x = 0%, 2%, 3%, 4%, 5% (mass fraction)) of common liquid precipitation phase composition and element Cu on its liquid phase precipitation formation regularity [6]. Manasijevic et al. examined the thermal and microscopic features of the piston alloys and found that changes in the concentrations of various alloying elements changed the composition and shape of the phases due to segregation and redistribution [7]. The microstructure of Al-13Si-4Cu-1Mg-2Ni alloy is composed of Al_3_Ni, Al_2_Cu, Mg_2_Si, Al_3_CuNi, Al_7_Cu_4_Ni, Al_5_Cu_2_Mg_8_Si_6_ and Al_9_FeNi. The size and shape of these phase changes are closely related to the solidification process. Thus, solidification control is very important to alter the microstructure of the alloy. However, the research on the influence of solidification cooling rate on the microstructure and properties of the alloy is still relatively little. This paper discusses the properties of the alloy under different solidification cooling rates, in pursuit of providing guidance for the design and manufacture of the piston alloy.

## 2. Experimental Methods and Processes

In the experimental study, an alloy model of Al-13Si-4Cu-1Mg-2Ni (wt.%) was prepared in an intermediate frequency induction melting furnace. Pure aluminum ingot blocks were added into the graphite crucible and were melted firstly, then the pieces of pure silicon, Al-Ni and Al-Cu intermediate alloys were added into the melt, respectively. Furthermore, mischmetal, rich in elements La and Ce, together with Al-P alloy as the modifier agents were added into the molten alloy. Hexachloroethane as the degassing agent was added at 720–740 °C and held for 15 min. Then Mg block was added under the melt completely to keep it from contacting oxygen and burning. After skimming off the slags covered on the melt surface, the clean melt was poured into the different molds with various cooling rates and solidified to the ingots.

**Figure 1:**
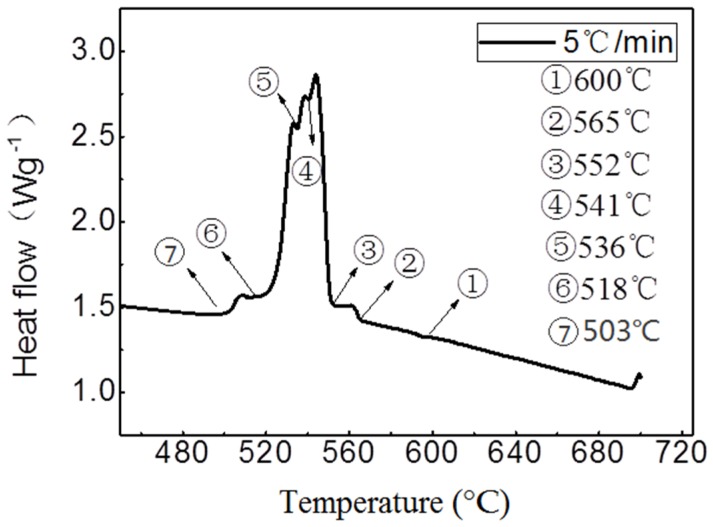
The DSC (differential scanning calorimetry) curve of the alloy with the cooling rate of 15 °C/s.

The cooling rate is calculated by recording the temperature and time point of solidification process with the temperature paperless recorder in different molds (sand mold, metal mold, copper mold with water cooling). The solidification temperature range is from 650 to 450 °C as demonstrated in Figure 1. Three sets of dates were tested for each sample and the approximations were obtained. The preparation methods of the test alloys with different cooling rates are shown in Table 1.

The composition of the alloy was measured by a direct-reading spectrometer (model Bruker Q4 Tasman). The results are shown in Table 2.

The as cast microstructures of the Al-13Si-4Cu-1Mg-2Ni alloy solidified with different cooling rates were analyzed using a Nikon EPIPHOT300 optical microscope (Nikon, Tokyo, Japan) and a Quanta 400F (FEI, Hillsboro, OR, USA) thermal field emission scanning electron microscope equipped with an INCA energy dispersive spectrometer. The phase compositions contained in the alloy prepared at different cooling rates were examined using an XRD-6000 X-ray diffract meter (Shimadzu, Kyoto, Japan). Brinell hardness tested with a HB3000 hardness tester (Shanghai shangcai testing machine Co. Ltd., Shanghai, China) has a load of 250 N. Vickers hardness load is 200 N with a WilsonV32D265 (Wilson, Duisburg, Germany). It was utilized to measure the hardness of the three ingot samples prepared at different cooling rates. The tensile strengths of the cast Al-13Si-4Cu-1Mg-2Ni alloy was tested with the Instron 1195 mechanical testing machine (Instron, Shanghai, China).

## 3. Results and Discussion

### 3.1. Microstructural Analysis of the Samples at Different Cooling Rates

**Figure 2:**
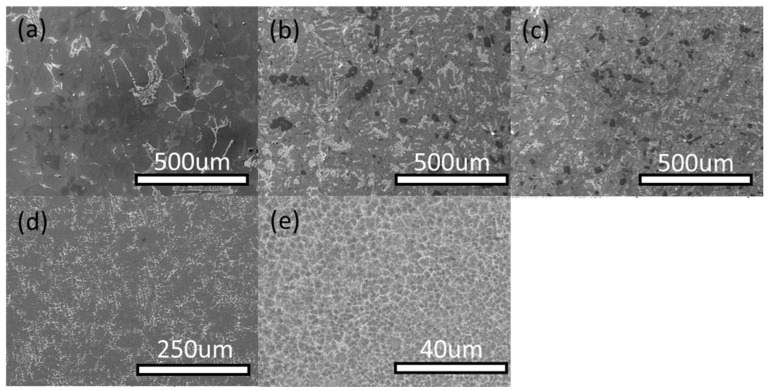
SEM analysis in the Al-13Si-4Cu-1Mg-2Ni alloy solidified with different cooling rates: (**a**) 0.15 °C/s; (**b**) 1.5 °C/s; (**c**) 15 °C/s; (**d**) 150 °C/s; (**e**) 1.5 × 10^5^ °C/s.

Figure 2 shows the microstructures of the model alloy solidified with different cooling rates. The solidification phase composition of alloy varies with cooling rate. The figure indicates that for cooling rates in the range of 0.15–1.5 × 10^5^ °C/s, the microstructure of the alloy turns to be noticeably refined as the cooling rate increases, and dark gray eutectic silicon changes from continuous small strips to small granules. The size of white Ni- and Cu-rich alloy phase becomes smaller, and the distribution of the phases is more uniform in the structure. When the cooling rate exceeds 1.5 × 10^5^ °C/s, a uniform granular α-Al grain formed, and the second phase which is small in size is distributed around the α-Al grain, because the second phase produced during solidification is too fast to grow.

It can be seen from Figure 2, as the cooling rate increases, the secondary dendrite arm spacing (SDAS) obviously decreases. This is due to the fact that with the increased cooling rate, both the critical nucleation radius and the nucleation activation energy decrease and the nucleation rate increases [8,9,10]. The high cooling rate results in higher temperature gradient at the leading edge of the solid–liquid interface, thereby leading to more heterogeneous nucleation and inhibiting the grain growth. The results of SDAS at different cooling rates are shown in Table 3.

The following classical relationship between the metal cooling rate and SDAS is derived from the theory of heat transfer and mass transfer:

*D* = *βv*^−1/3^ (1)

where *β* is a constant related to the alloy composition and cooling conditions. Using power law regression analysis, a quantitative relationship between the cooling rate (*v*) and the SDAS (*D*) of the model alloy can be obtained by fitting the data:

*D* = 47.126*v*^−1/3^ (2)

**Figure 3:**
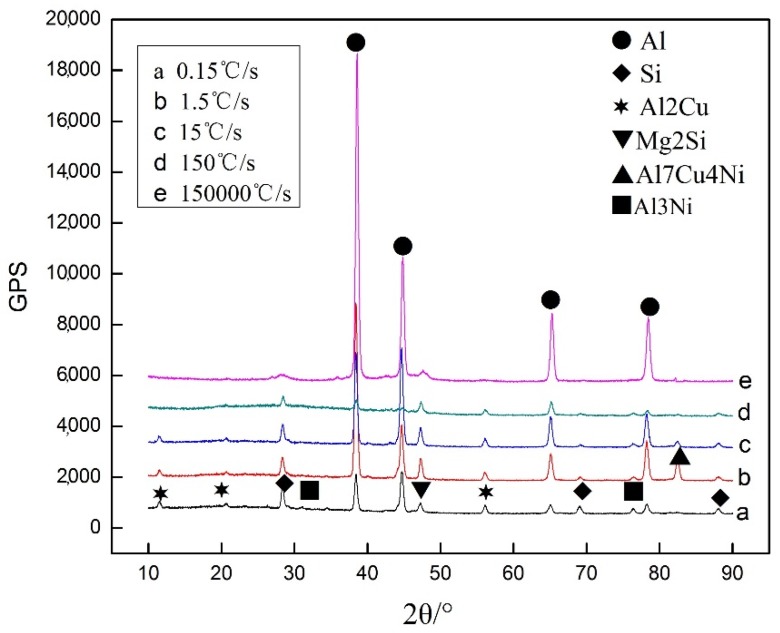
XRD analysis in the Al-13Si-4Cu-1Mg-2Ni alloy solidified with different cooling rates. (**a**) 0.15 °C/s; (**b**) 1.5 °C/s; (**c**) 15 °C/s; (**d**) 150 °C/s; (**e**) 1.5 × 10^5^ °C/s.

**Figure 4:**
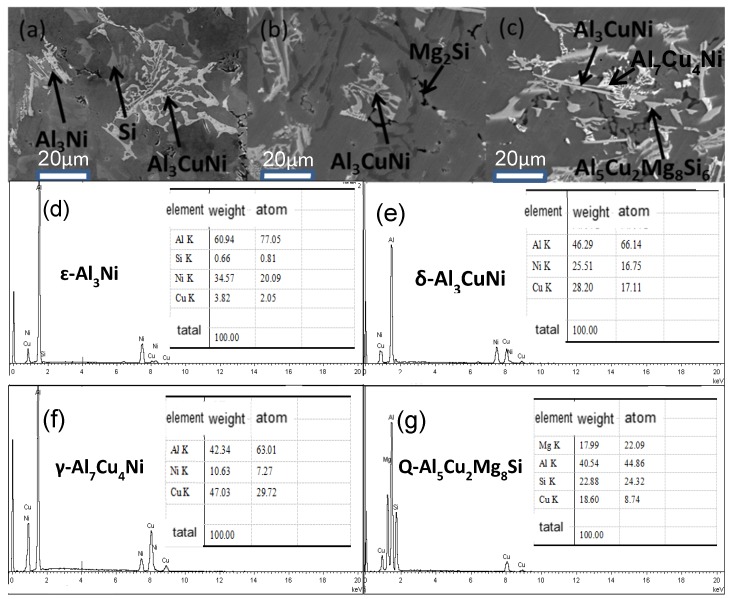
SEM analysis in the Al-13Si-4Cu-1Mg-2Ni alloy solidified with different cooling rates: (**a**) 0.15 °C/s; (**b**) 1.5 °C/s; (**c**) 15°C/s; (**d**) EDS of ε-Al_3_Ni; (**e**) EDS of δ-Al_3_CuNi; (**f**) EDS of γ-Al_7_Cu_4_Ni; (**g**) EDS of Q-Al_5_Cu_2_Mg_8_Si_6._

Figure 3 shows the XRD patterns of Al-13Si-4Cu-1Mg-2Ni alloy at different cooling rates. The figure reveals that the composition of the alloy is similar to that of solidification under different cooling rates, but the content of each phase is different. There is ε-Al_3_Ni in the XRD analysis of a sample at cooling rate of 0.15 °C/s, and there is almost no ε-Al_3_Ni phase in the alloy when the cooling rate is increased greatly, as demonstrated in the SEM analysis in Figure 4. It also indicates that there are almost no other alloying phases forms in the Al alloy during solidification with the cooling rate of 1.5 × 10^5^ °C/s except for α-Al phase. Because α-Al precipitated from under-cooling melt, the residual melt did not have enough time to form other big second alloying phases, and therefore nanocrystal exists in the alloy.

Figure 4 presents an enlarged view of the SEM micrograph of the model alloy at: (a) 0.15 °C/s (b) 1.5 °C/s (c) 15 °C/s. Energy dispersive spectroscopy analysis of the white phase has confirmed it to be δ-Al_3_CuNi phase and γ-Al_7_Cu_4_Ni phase. It has been reported that the major strengthening formed during the solidification process in the piston alloy are δ-Al_3_CuNi phase and γ-Al_7_Cu_4_Ni phase [7].

Figure 4a suggests that there are ε-Al_3_Ni phases in the solidified ingot at the subcooling rate, and there are few black M-Mg_2_Si phases. With the increase in the cooling rate, the black M-Mg_2_Si phase rises slightly, and the δ and γ phases changed from large meshes and clusters to smaller meshes and Chinese script features. At a cooling rate of 15 °C/s, there are Q-Al_5_Cu_2_Mg_8_Si_6_ phases in the microstructure of the ingot, and most of them are around the δ phase. However, no Q phase is found in the ingot microstructure at a cooling rate of 0.15 °C/s because Mg_2_Si is preferentially precipitated. The δ and Q phases both increase with rising cooling rate. Belov et al. studied the phase diagrams of the Al-Cu–Fe–Mg–Ni–Si system of aluminum piston alloys [11], the Al-Si-Cu-Mg-Ni alloy reported in the literature is the same as the alloy system studied in this paper, so the DSC curve is similar, which is shown in the literature: the Q phase formed through L → (Al) + (Si) + θ + Q + γ at about 503 °C. When the cooling rate increases, the temperature decreases so fast that the high temperature precipitates did not have enough time to precipitate. In this model alloy, the morphology of the second phase is characterized by irregularity, which is generally attributed to the alterations in the temperature gradient and the degree of overcooling and composition changes during the solidification of the alloy. It is the cooling rate of the alloy in the solidification stage that influences the microstructural morphology of the alloy; it allows the secondary phase of the alloy to have a significant change in size and morphology [11,12,13,14]. When the cooling rate is accelerated, the energy fluctuation is larger and the nucleation rate increases. For the crystal grain, the liquid-solid phase transitions take place even before the crystal grain grows up, thus producing fine grains and a deviation from the equilibrium state.

**Figure 5:**
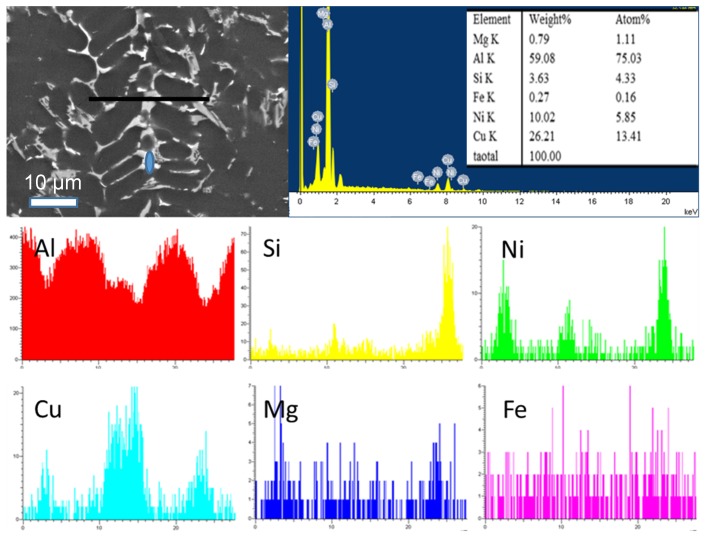
SEM analysis with the cooling rate of 150 °C/s.

**Figure 6:**
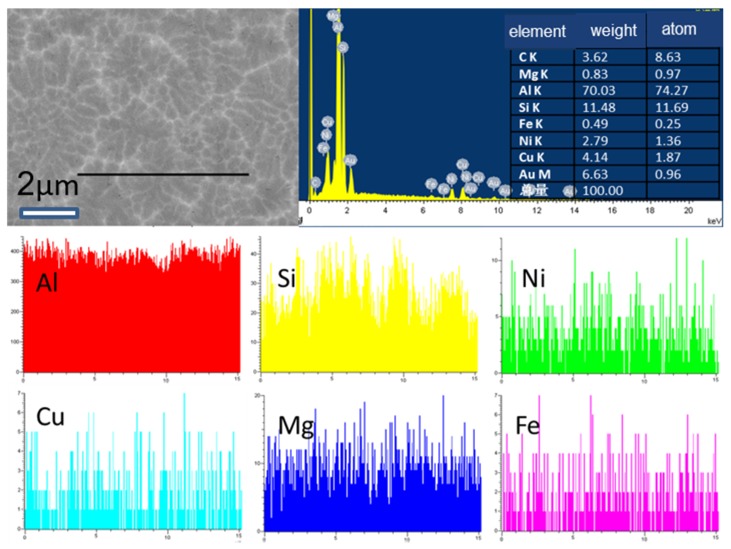
SEM analysis in the Al-13Si-4Cu-1Mg-2Ni alloy solidified with cooling rate of 1.5 × 10^5^ °C/s.

Figure 5 shows a high-magnification scanning microscopy image for the cooling rate of 150 °C/s and a line scanning elemental profile. In the figure, the gray-colored region is the aluminum matrix, and the fine white phase may be the γ-Al_7_Cu_4_Ni through EDS and XRD in Figure 6. From the line-scan distribution pattern, the complex compounds produce in a uniform symmetrical growth manner during the α-Al solidification process due to the excessive cooling rate. In the meantime, when the cooling rate continues to increase to 1.5 × 10^5^ °C/s (Figure 5), the SDAS is too small to distinguish gray-colored regions from bright white-colored regions, and one can only see that the elements of the alloy are evenly distributed to form a solid solution.

**Figure 7:**
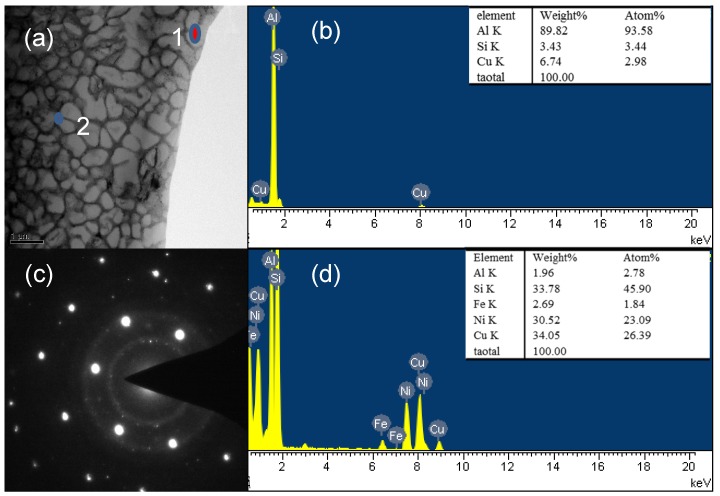
TEM analysis with the cooling rate 1.5 × 10^5^ °C/s.

Figure 7 shows the TEM image and EDS analysis of the sample at the cooling rate of 1.5 × 10^5^ °C/s. It is clearly demonstrated that something like a polycrystalline ring or an amorphous ring is formed around the [110] zone axis of Al matrix. EDS analysis of the matrix shows a small amount of Si and Cu in the Al matrix, whereas the so-called grain boundaries are alloying elements in the states. Such crystals are called nanocrystals because the matrix Al content is too high to completely form amorphous. 

### 3.2. Analysis of the Mechanical Properties of the Samples at Different Cooling Rates

**Figure 8:**
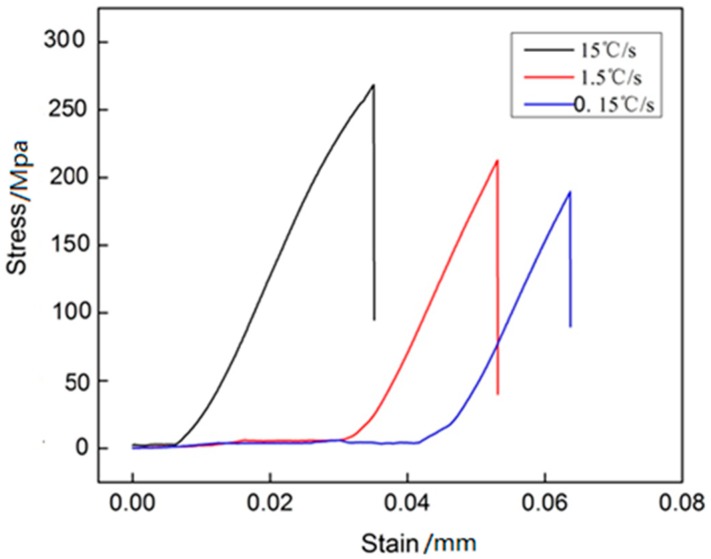
The stress strain curve of different cooling rate of the alloy.

As seen from Table 3, with the increase in cooling rate, the SDAS changes rather significantly. Table 4 lists the test results of the cast model alloy solidified with different cooling rates. The results show that as the solidification cooling rate accelerated, hardness changes from 80.9 to 125.7 HB and the micro hardness changes from 130 to 195 HV. Figure 8 shows the stress strain curve of different cooling rate of the alloy.

Tensile strength at room temperature has also increased significantly by 49.2% and the elongation increased from 1.6% to 2.8% since as the cooling rate increases, the grains become considerably finer, and the various second phases dispersed more evenly, thereby giving rise to the dual effect of fine grain strengthening and second phase strengthening.

It is evident from Figure 8 that the tensile strength is the point at which the premature failure occurs. This means that the improved tensile strength at high cooling rates is related to the refinement of secondary phases that delays the premature failure. Both the strengthening by fine phases and the delay of premature failure contribute to the improved mechanical properties at high cooling rates. The fine alloy phase size is the main reason for the reinforcement. With the strengthening in these manners, there are improvements in the strength of the alloy material, the processing performance of the material and the toughness of the alloy material.

## 4. Conclusions

By comparing the different cooling rate, the microstructure, hardness and tensile strength of the alloy all changed obviously. The main findings are summarized as follows:

1. With the increase in the solidification cooling rate from 0.15 to 1.5 × 105 °C/s, the SDAS of Al-13Si-4Cu-1Mg-2Ni alloy is refined from 84.1 µm to 0.82 µm. With the increase in the solidification cooling rate from 0.15 to 1.5 × 105 °C/s, the main strengthening phases, δ-Al_3_CuNi phase and γ-Al_7_Cu_4_Ni phase, increased and the shape changed from the larger continuous mesh and long chains into smaller clusters and granules. The formation of Q-Al_5_Cu_2_Mg_8_Si_6_ phase gradually increases while the ε-Al_3_Ni phase decreases. There is a rapid change in alloy structure, from different polymetallic phases to a single nanocrystal.
2. The mechanical properties of the as cast Al-13Si-4Cu-1Mg-2Ni alloy at room temperature including hardness, tensile strength and elongation were noticeably improved due to the effect of fine grain strengthening, fine second phase strengthening, and dispersion strengthening. The hardness increased from 80.9 to 125.7 HB, the room temperature tensile strength enhanced from 189.3 to 282.5 MPa and the elongation at break increased from 1.6% to 2.8%. Further increase in the cooling rate leads to the micro hardness increases gradually from 131.2 to 195.6 HV.

## Figures and Tables

**Table 1:** The preparation methods of different cooling rate alloys.

Cooling Rate °C/s	Casting Mold	Mold Size	Type of Furnace	Cooling Rate Measurement
0.15	Sand mold	Ø50 mm Cylinder length: 150 mm	induction melting furnace	paperless recorder thermocouple GZJ-800G
1.5	Metal mold	Ø55 mm Cylinder length: 150 mm	induction melting furnace	paperless recorder thermocouple GZJ-800G
15	copper mold with water cooling	Ø50 mm Cylinder length: 150 mm	induction melting furnace	paperless recorder thermocouple GZJ-800G
150	copper mold with water cooling	Ø4 mm Cylinder length: 10 mm	vacuum induction melting furnace	paperless recorder thermocouple GZJ-800G
150,000	melt-spinning	strip	vacuum induction melting furnace	analog computation

**Table 2:** Element content in the test alloy (wt.%).

Material	Si	Cu	Mg	Ni	Al
Al-13Si-4Cu-1Mg-2Ni	12.76	4.03	1.06	2.17	balance

**Table 3:** SDAS of the alloy ingot at different cooling rates.

Cooling Rates/°C/s	0.15	1.5	15	150	1.5 × 10^5^
SDAS/μm	84.1	43.9	24.8	13.5	0.82

**Table 4:** Mechanical properties of the cast model alloy solidified with different cooling rates.

Colling Rate °C/s	Hardness		Tensile Strength/MPa	Elongation/%
0.15	80.9 HB		189.3	1.6
1.5	118.6 HB		213.3	2.2
15	125.7 HB	108.6 HV matrix	282.5	2.8
		131.2 HV phases		
150	150.4 HV		-	-
15,000	195.6 HV		-	-
